# Reduced expression of cathepsin F predicts poor prognosis in patients with clear cell renal cell carcinoma

**DOI:** 10.1038/s41598-024-64542-2

**Published:** 2024-06-12

**Authors:** Xin Zhou, Huayan Chen, Dong Huang, Guixian Guan, Xiaoli Ma, Weiming Cai, Jing Liao, Tangming Guan

**Affiliations:** 1 School of Chemistry and Environmental Science, Guangdong Ocean University, Zhanjiang, 524088 China; 2 Department of Pharmacy, Affiliated Hospital of Guangdong Medical University, Guangdong, 524001 China; 3 Department of Gastroenterology, Heyuan People’s Hospital, Heyuan, China; 4 Department of Obstetrics and Gynecology, Affiliated Hospital of Guangdong Medical University, Guangdong, 524001 China

**Keywords:** Cathepsin F function, Prognosis, Renal cell carcinoma biomarker, Tumor microenvironment, Cancer, Computational biology and bioinformatics, Biomarkers, Oncology, Risk factors

## Abstract

Abnormalities in the extracellular matrix (ECM) play important roles in the regulation and progression of clear cell renal cell carcinoma (ccRCC). The cysteine cathepsin is one of the major proteases involved in ECM remodeling and has been shown to be aberrantly expressed in multiple cancer types. However, the clinical significance and biological function of distinct cysteine cathepsins in ccRCC remain poorly understood. In this study, several bioinformatics databases, including UALCAN, TIMER, GEPIA and the Human Protein Atlas datasets, were used to analyze the expression and prognostic value of different cysteine cathepsin family members in ccRCC. We found that the expression level of CTSF was downregulated in tumor tissues and closely related to the poor survival of ccRCC patients. Further in vitro experiments suggested that CTSF overexpression suppressed the proliferation and migration of ccRCC cells. Moreover, the expression of CTSF was shown to be associated with several immune-infiltrating cells and immunomodulators in ccRCC. These results indicated that CTSF might be a promising diagnostic and prognostic marker in ccRCC.

## Introduction

Kidney cancer is one of the top 10 cancer types diagnosed in men (2.7% of total cases)^1^. More than 430,000 new patients were diagnosed with this disease, and more than 170,000 kidney cancer patients died from the disease worldwide in 2020^1^. Among these subtypes, clear cell renal cell carcinoma (ccRCC) is the most common histological subtype and accounts for ~ 70% of all kidney cancers^2^. However, early diagnosis of ccRCC is difficult, and metastases often occur before the primary tumor is detected. Therefore, the identification of new diagnostic biomarkers and therapeutic targets is urgently needed for the diagnosis, treatment, and prognostic assessment of ccRCC.

Numerous studies have demonstrated that aberrations in the extracellular matrix (ECM) play important roles in the regulation and progression of ccRCC^3,4^. Cysteine cathepsins, the major proteases involved in ECM remodeling, have been shown to be aberrantly expressed in multiple cancer types and to participate in the tumorigenesis, progression and metastasis of these cancers^5,6^. However, cysteine cathepsins can demonstrate either tumor-promoting or tumor-suppressive effects depending on the cancer type^5,6^. To date, eleven human cysteine cathepsins (B, C, H, F, K, L, O, S, L2/V, W, and Z) have been identified in mammalian cells. They are involved in numerous important pathophysiological processes, such as protein degradation, autophagy, antigen presentation, immune cell activation and cellular signal transduction^5,7,8^. However, the clinical significance and biological function of distinct cysteine cathepsins in ccRCC remain poorly understood.

In this study, the expression and prognostic value of different cysteine cathepsin family members in ccRCC were explored via diverse databases, including the University of ALabama at Birmingham Cancer Data Analysis (UALCAN), Tumor Immune Estimation Resource (TIMER), Gene Expression Profiling Interactive Analysis (GEPIA) and Human Protein Atlas datasets. In particular, CTSF was downregulated in tumor tissues, which is closely related to the poor survival of ccRCC patients. Moreover, the overexpression of CTSF significantly reduced the proliferation and migration ability of ccRCC cells. Finally, we also found that the expression of CTSF was associated with the infiltration of several immune-infiltrating cells and immunomodulators in ccRCC. Our findings revealed the noticeable role of CTSF in tumorigenesis and progression and indicated that CTSF may contribute to the regulation of the tumor immune microenvironment in ccRCC.

## Results

### Aberrant expression of the cysteine cathepsin family in KIRC patients

**Figure 1:**
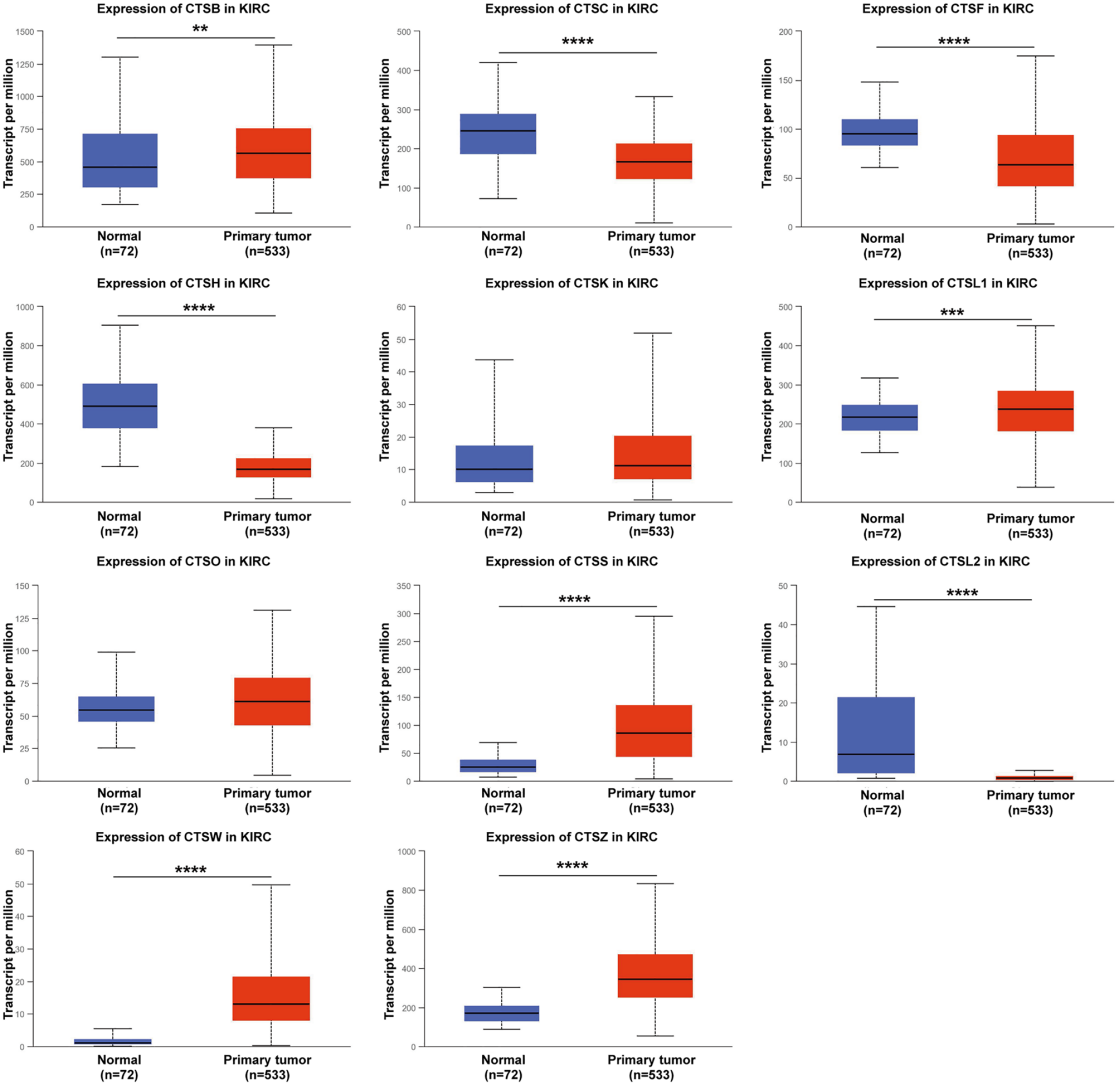
mRNA expression levels of the cysteine cathepsin family members in KIRC and normal tissues according to the UALCAN database. Red, primary tissues of KIRC. Blue, normal tissues. ** *p* < 0.01, *** *p* < 0.001, **** *p* < 0.0001.

Eleven human cysteine cathepsins have been identified in mammalian cells. First, we compared the mRNA expression of cysteine cathepsins in kidney renal clear cell carcinoma (KIRC) samples with that in normal samples by using the UALCAN database. As shown in Fig. 1, compared with those in normal kidney tissues, the expression levels of CTSC, CTSF, CTSH and CTSL2 (CTSV) were significantly downregulated, while the expression levels of CTSB, CTSL1 (CTSL), CTSS, CTSW and CTSZ were significantly upregulated. We also utilized the TIMER database to analyze the relative expression levels of cysteine cathepsins in KIRC (Fig. S1). Interestingly, these results were consistent with those obtained from the UALCAN database.

**Figure 2:**
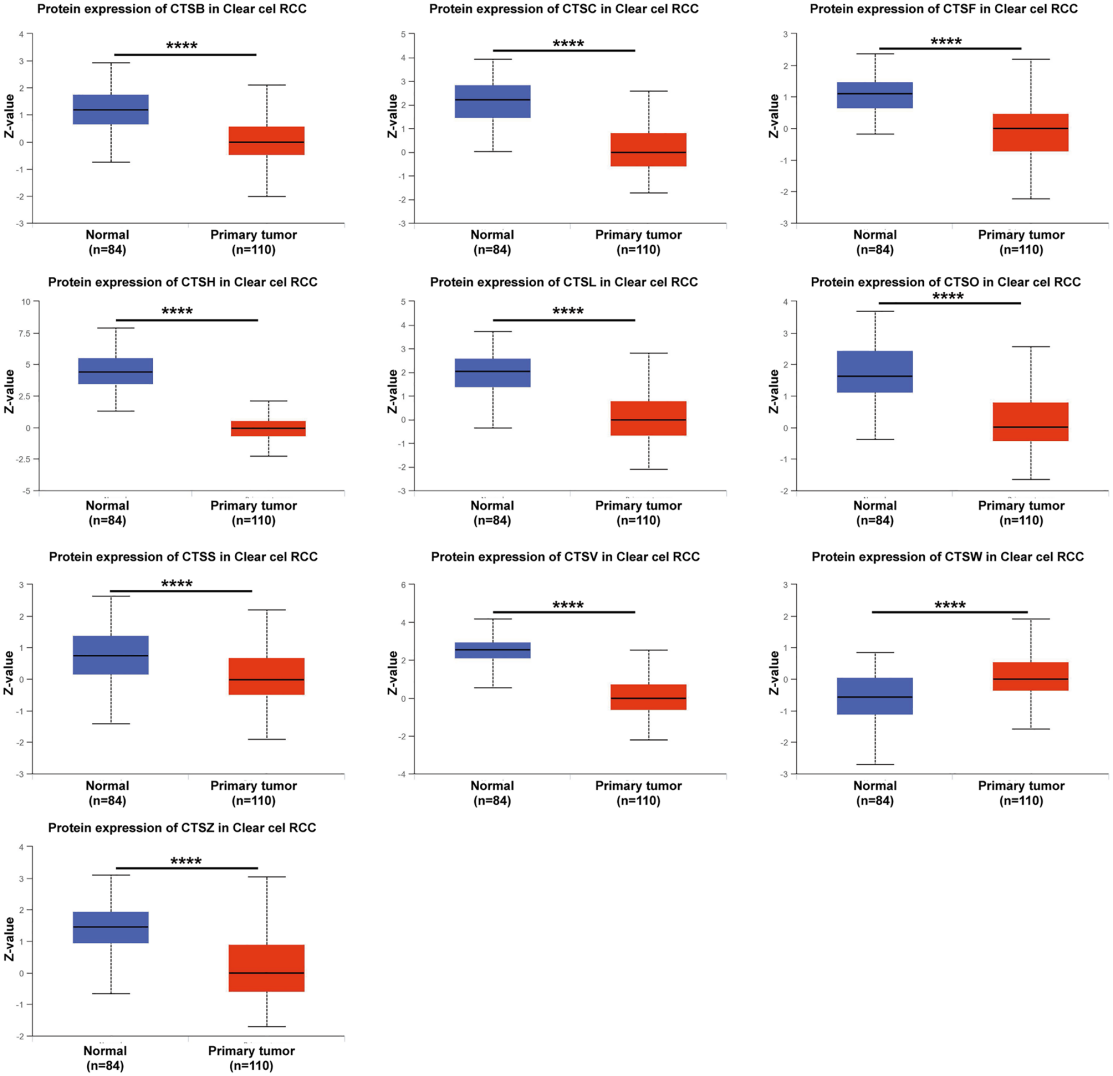
Protein expression levels of the cysteine cathepsin family members in KIRC and normal tissues according to the UALCAN database. Red, primary tissues of KIRC. Blue, normal tissues. **** *p* < 0.0001.

After analyzing the mRNA expression of the cysteine cathepsins in KIRC, we next explored the protein expression levels of cysteine cathepsins using the CPTAC database. As shown in Fig. 2, the protein levels of CTSB, CTSC, CTSF, CTSH, CTSL, CTSO, CTSS, CTSL2 (CTSV) and CTSZ in KIRC tissues were lower than those in normal kidney tissues, while the CTSW protein level was significantly upregulated in KIRC tissues. Taken together, these results suggested that the expression of CTSF, CTSH and CTSL2 was downregulated in KIRC, while the expression of CTSW was upregulated.

### Prognostic value of cysteine cathepsins in KIRC patients

**Figure 3:**
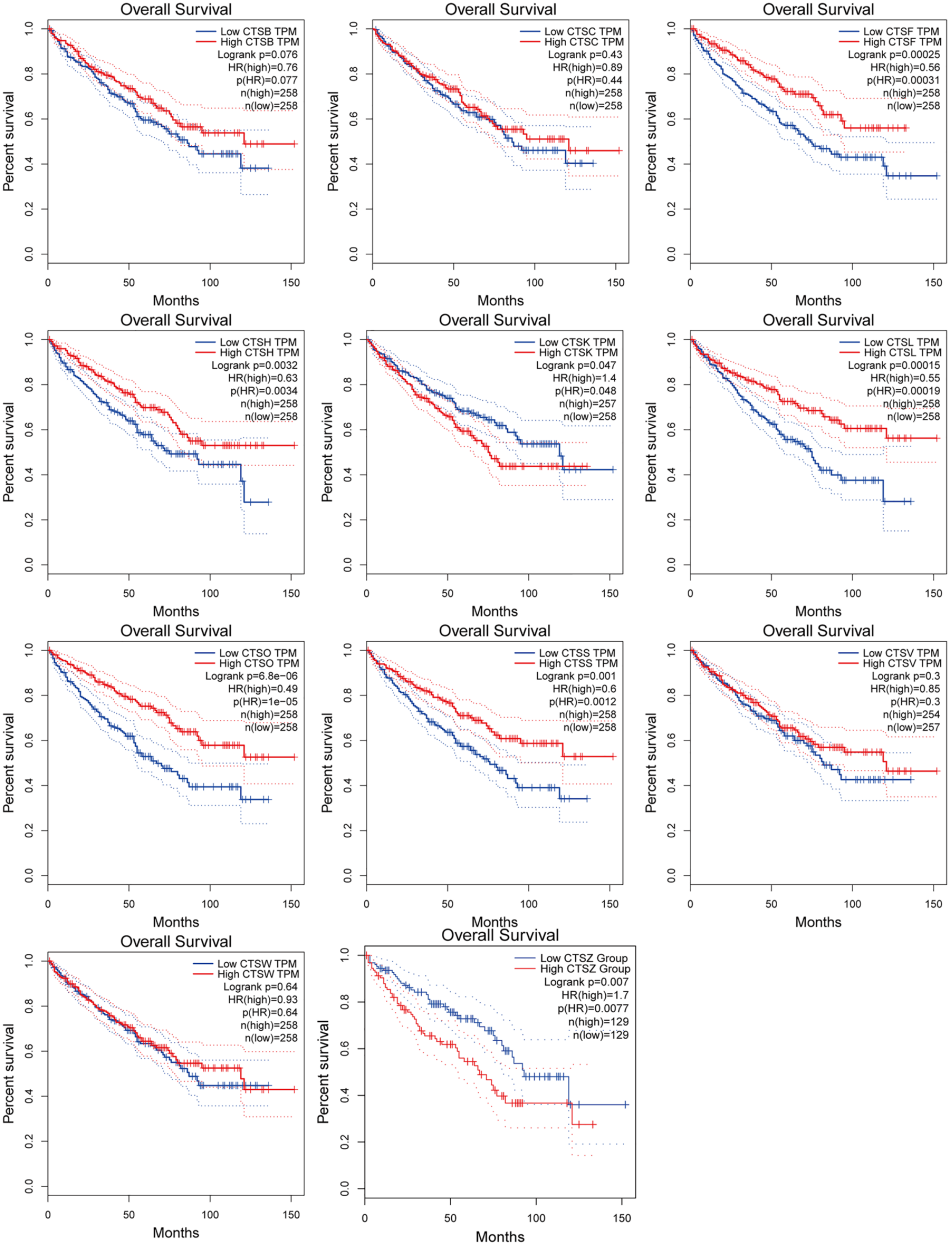
The overall survival of KIRC patients based on the cysteine cathepsin family genes mRNA expression according to the GEPIA database. HR refers to hazard ratio. Data were analyzed using the Kaplan–Meier Plotter.

We further explored the prognostic value of cysteine cathepsins for the survival of patients with KIRC using the GEPIA database. As shown in Figs. 3 and S2, high mRNA expression of CTSF (OS: HR = 0.56, log-rank *P* = 0.00025; DFS: HR = 0.52, log-rank *P* = 0.00057), CTSL (OS: HR = 0.55, log-rank *P* = 0.00015; DFS: HR = 0.55, log-rank *P* = 0.0014) and CTSO (OS: HR = 0.49, log-rank *P* = 6.8e − 06; DFS: HR = 0.61, log-rank *P* = 0.0076) were related to favorable overall survival (OS) and disease-free survival (DFS) in KIRC patients. High mRNA expression of CTSK (OS: HR = 1.4, log-rank *P* = 0.047; DFS: HR = 1.8, log-rank *P* = 0.03) and CTSZ (OS: HR = 1.7, log-rank *P* = 0.007; DFS: HR = 1.5, log-rank *P* = 0.022) was significantly correlated with poor OS and PFS in KIRC patients. In addition, higher mRNA expression of CTSH and CTSS was related to favorable OS but was not related to PFS in KIRC patients. However, the mRNA expression of CTSB, CTSC, CTSV and CTSW was not related to the prognosis of KIRC patients (Figs. 3, S2).

**Figure 4:**
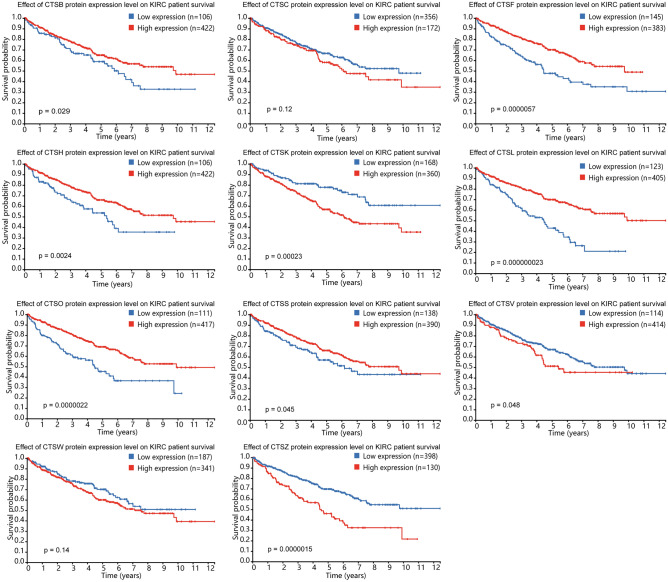
The overall survival of KIRC patients based on the cysteine cathepsin family genes expression according to the Human Protein Atlas database.

We also utilized the Human Protein Atlas database to explore whether the expression of cysteine cathepsins was associated with KIRC patient survival. As shown in Fig. 4, high expression of CTSB, CTSF, CTSH, CTSL, CTSO and CTSS was related to favorable survival in KIRC patients. High CTSK, CTSV and CTSZ expression was significantly correlated with poor survival in KIRC patients.

Taken together, these results revealed that CTSF, CTSL, CTSO, CTSK, and CTSZ are prognostic factors. Among them, high expression of CTSF, CTSL and CTSO was favorable for KIRC, while high expression of CTSK and CTSZ was unfavorable for KIRC.

### CTSF suppressed the proliferation and migration of ccRCC cells

**Figure 5:**
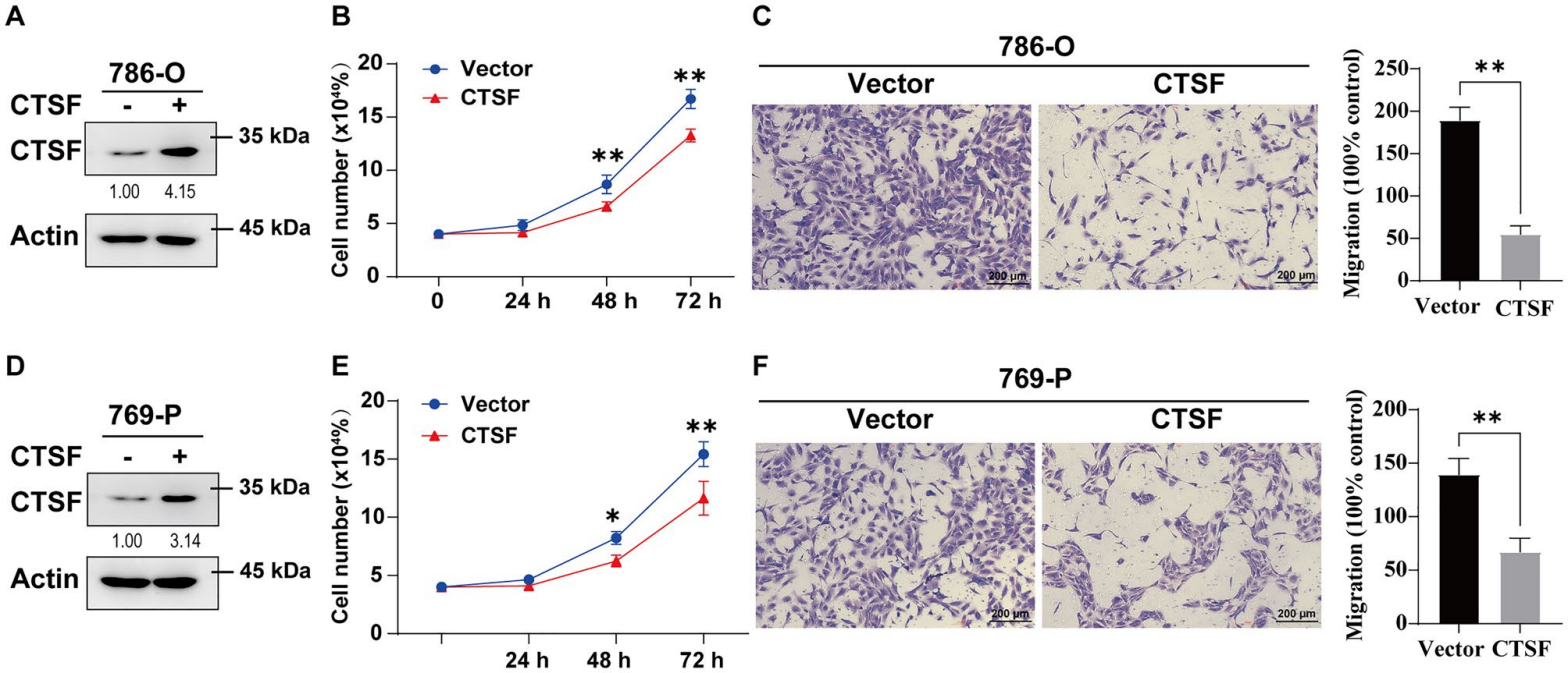
CTSF suppresses the proliferation and migration of ccRCC cells. (**A**) Western blotting results showed that CTSF was highly expressed in 786-O cells following transfection with CTSF expression plasmid compared with pCMV-HA control vector. (**B**, **C**) The proliferation and migration abilities of the cells described in (**A**) were measured via cell proliferation and transwell migration assays. The results are presented as the mean ± s.d. from three independent experiments; *p < 0.05, **p < 0.01, CTSF vs Vector. (**D**) Western blotting results showed that CTSF was highly expressed in 769-P cells following transfection with CTSF expression plasmid compared with pCMV-HA control vector. (**E**, **F**) The proliferation and migration abilities of the cells described in (**D**) were measured via cell proliferation and transwell migration assays. The results are presented as the mean ± s.d. from three independent experiments; *p < 0.05, **p < 0.01, CTSF vs Vector.

Based on the above expression and prognostic results for cysteine cathepsins, we found that the mRNA and protein expression levels of CTSF were downregulated only in KIRC tissues and closely related to the unfavorable prognosis (OS and DFS) of patients with KIRC. These results indicated that CTSF might be an ideal therapeutic target and a promising diagnostic marker in KIRC. In addition, the mRNA expression levels of CTSF had a trend to lower expression in more advanced cancer stages and higher grades (Fig. S3). Thus, we used cell experiments to further explore the function of CTSF in ccRCC. As shown in Fig. 5, the overexpression of CTSF significantly reduced the proliferation and migration ability of 786-O and 769-P cells. These findings suggested that CTSF might be a tumor suppressor in ccRCC.

### PPI network and functional enrichment analysis of CTSF-related genes

**Figure 6:**
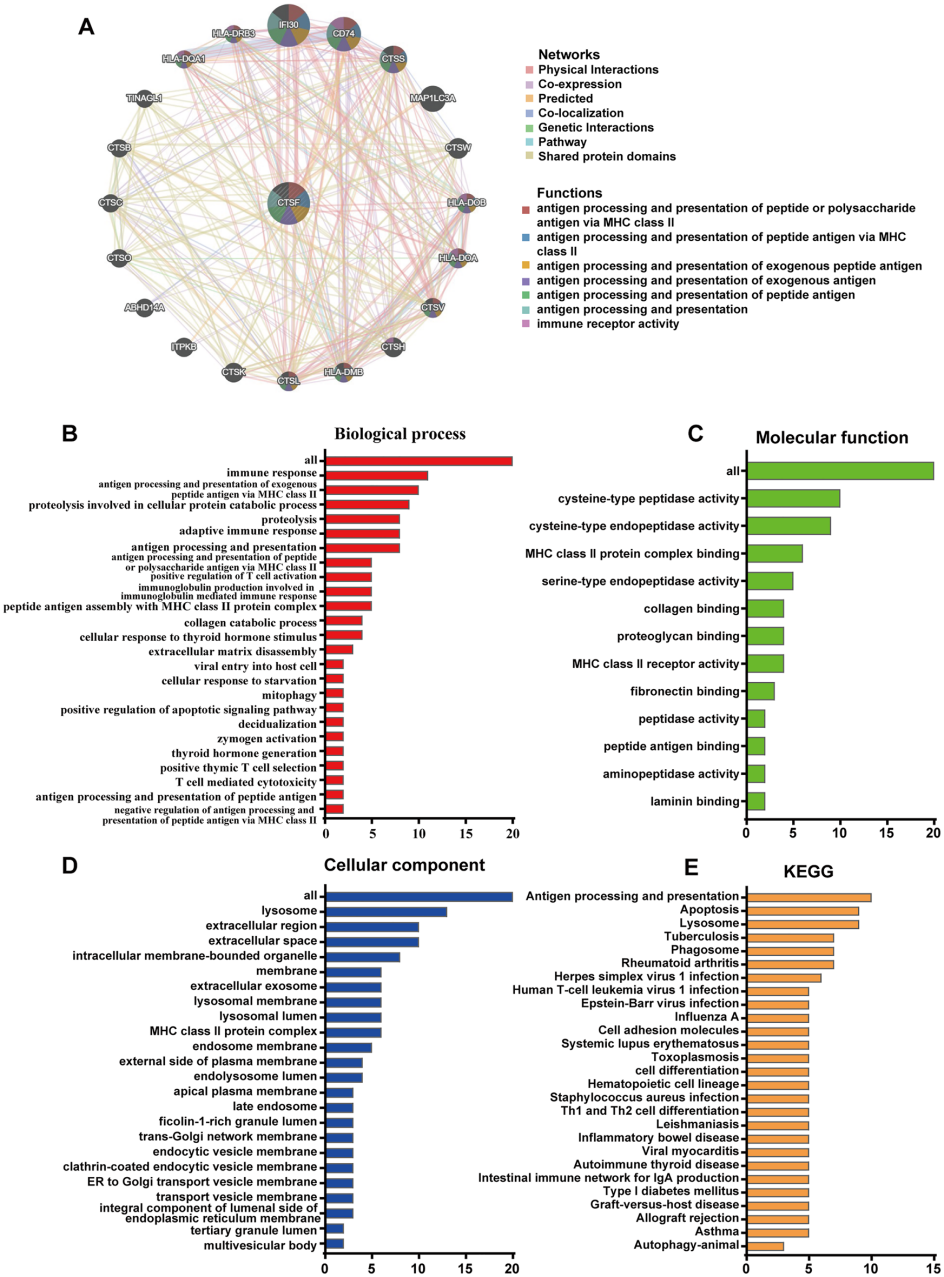
Predicted functions and pathways of the CTSF and the 20 most related genes. (**A**) The gene–gene interaction network of CTSF was constructed using GeneMania. (**B**–**D**) Biological process (**B**), molecular functions (**C**) and cellular components (**D**) of CTSF interaction partners were predicted by GO functional enrichment analysis. (**E**) KEGG pathway analysis on CTSF interaction partners was shown.

To better understand the biological functions of CTSF in KIRC, we first constructed a gene‒gene interaction network for CTSF and the 20 most related genes using GeneMANIA. The 20 genes related to CTSF were IFI30, CD74, CTSS, MAP1LC3A, CTSW, HLA-DOB, HLA-DOA, CTSV, CTSH, HLA-DMB, CTSL, CTSK, ITPKB, ABHD14A, CTSO, CTSC, CTSB, TINAGL1, HLA-DQA1 and HLA-DRB3 (Fig. 6A).

Moreover, we performed GO and KEGG enrichment analyses to explore the functional annotation and involved pathways of CTSF-related proteins in the DAVID database. As shown in Fig. 6B–D, proteins encoded by these 20 genes were classified into three categories: biological process, molecular function and cellular component. First, we found that the CTSF-related proteins were involved in the immune response, antigen processing and presentation of exogenous peptide antigens via MHC class II, proteolysis involved in cellular protein catabolic processes, antigen processing and presentation, the adaptive immune response and proteolysis. Moreover, the genes were enriched mainly in the molecular function terms cysteine-type peptidase activity, cysteine-type endopeptidase activity, MHC class II protein complex binding and serine-type endopeptidase activity. Moreover, in terms of cellular components, these CTSF-related proteins were significantly enriched in the lysosome, extracellular space, extracellular region and intracellular membrane-bound organelles. In addition, KEGG analysis revealed that the CTSF-related proteins were enriched mainly in the antigen processing and presentation, lysosome and apoptosis pathways (Fig. 6E).

### The expression of CTSF was associated with TILs and immunomodulators in ccRCC

**Figure 7:**
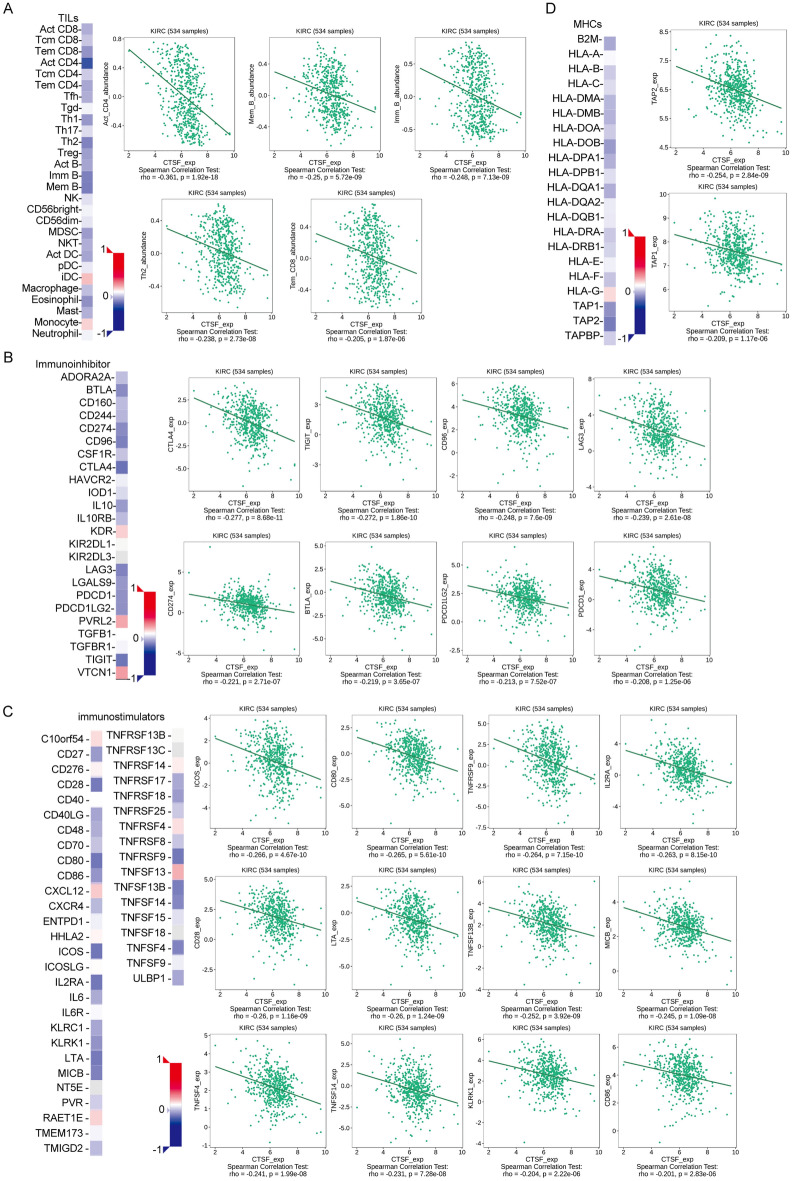
The expression of CTSF was associated with TILs and immunomodulators in ccRCC. (**A**) Correlation between the expression of CTSF and the abundance of TILs in ccRCC available at TISIDB database. (**B**–**D**) Correlation between CTSF expression and immunoinhibitors (**B**), immunostimulators (**C**) and MHCs (**D**) in ccRCC available at TISIDB database.

Numerous studies have demonstrated that MHC-mediated antigen presentation is critical for the induction of immune responses. The CTSF-related proteins were mainly involved in the immune response, antigen processing and presentation, and the adaptive immune response. We next examined the potential correlation between CTSF expression and tumor-infiltrating lymphocytes (TILs) in KIRC patients. As shown in Fig. 7A, we found that the CTSF expression level was strongly correlated with activated CD4 T cells (Act CD4), memory B cells (Mem B), immature B cells (Imm B), type 2 T helper cells (Th2), eosinophils and effector memory CD8 T cells (Tem CD8) (Spearman’s correlation coefficients were all <  − 0.2).

In addition, emerging evidence also suggests that immunomodulators play an important role in cancer immunotherapy^9–11^. We further investigated the relationship between CTSF and common immunomodulators, 22 immunoinhibitors, 42 immunostimulators, and 21 MHC molecules, in KIRC using the TISIDB database. As shown in Fig. 7B, the most relevant immunoinhibitors correlated with the expression of CTSF in KIRC were CTLA4, TIGIT, CD96, LAG3, CD274, BTLA, PDCD1LG2 and PDCD1 (Spearman’s correlation coefficients were all <  − 0.2). Moreover, the most relevant immunostimulators correlated with the expression of CTSF in KIRC were ICOS, CD80, TNFRSF9, IL2RA, CD28, LTA, TNFSF13B, MICB, TNFSF4, TNFSF14, KLRK1 and CD86 (Spearman’s correlation coefficients were all <  − 0.2) (Fig. 7C). Moreover, TAP2 and TAP1 were the most relevant MHC molecules correlated with the expression of CTSF in KIRC (Spearman’s correlation coefficients were all <  − 0.2) (Fig. 7D). These findings suggest that CTSF is associated with the regulation of immune interactions and might predict the response to immunotherapy in ccRCC patients.

## Discussion and conclusion

In recent years, an increasing number of studies have shown that abnormal ECM regulation plays a critical role in the development of ccRCC^3,4^. Human cysteine cathepsins, the major proteases involved in ECM remodeling, have been reported to participate in the tumorigenesis, progression and metastasis of various cancer types^5,6^. For example, CTSB-mediated cleavage of disabled-2 cells could regulate TGF-β-induced autophagy, thereby promoting chemotherapeutic resistance and tumor metastasis^12^. During squamous cell carcinomas (SCCs), CTSC expression by dermal/stromal fibroblasts and bone marrow-derived cells can regulate the progression to malignancy and overt SCC development^13^. Song et al. reported that CTSF might have an antitumor effect by regulating the immune response in non-small cell lung cancer^14^. In prostate cancer, it was reported that CTSH-mediated processing of talin might promote cancer cell progression by affecting integrin activation and adhesion strength^15^. CTSK inhibition could enhance anticancer drug sensitivity through USP27x-mediated upregulation of Bim via the downregulation of rapamycin^16^. In endometrial cancer, CTSL expression was shown to be correlated with the expression of the growth regulatory genes Ki-67, cyclin B1, MYBL2, p21/WAF, and HER2 receptor tyrosine kinase^17^. Hu et al. reported that depletion or inhibition of CTSO could increase tamoxifen sensitivity in ERα-positive breast cancer through regulation of BRCA1^18^. Upregulation of the lysosomal protease CTSS in human colorectal cancer (CRC)-associated fibroblasts (CAFs) enhances antigen cross-presentation, thereby suppressing tumor-specific T-cell function in human CRC^19^. CTSV was proven to suppress GATA3 expression in ER-positive breast cancers by facilitating its turnover via the proteasome^20^. High expression of CTSZ was proven to promote cancer cell invasion and growth via the Arg-Gl-Asp (RGD) motif, engagement of integrins, and subsequent activation of FAK-Src signaling in cancer cells^21^. Although a few cysteine cathepsins have been shown to participate in tumorigenesis and progression, the distinct roles of cysteine cathepsins in ccRCC remain poorly understood. In this study, the gene expression and prognostic value of different cysteine cathepsin family members in ccRCC were analyzed.

For the first time, we analyzed and summarized the mRNA expression levels of eleven cysteine cathepsins in ccRCC by using the UALCAN and TIMER databases. Similarly, compared with those in normal kidney tissues, the mRNA expression levels of CTSF, CTSH and CTSL2 (CTSV) in ccRCC tissues were significantly downregulated, while the expression levels of CTSB, CTSL, CTSS, CTSW and CTSZ were significantly upregulated. However, we found that the mRNA expression data for CTSF, CTSH, CTSL2 and CTSW were consistent with the subsequent protein expression data for cysteine cathepsins. This might be due to the small sample size, differing study designs and other clinical factors. Therefore, a well-designed and large-scale clinical study is needed to explore the relationship between the expression levels of cysteine cathepsins and ccRCC.

Currently, accumulating evidence has revealed that cysteine cathepsins are prognostic and predictive factors in human cancer^5,6^. For instance, in breast cancer, high CTSB and CTSL levels in the primary tumor were associated with poor DFS and OS^22^, and increased CTSK levels were associated with a high incidence of lymphatic spread and poor survival in patients with oral squamous cell carcinoma^23^. High serum CTSS levels are associated with favorable survival in lung cancer patients^24^, and a loss of CTSZ at the invasion front is associated with tumor progression and poorer OS in colorectal carcinoma patients^25^. Although the role of cysteine cathepsins in the tumorigenesis and prognosis of several cancers has been partially confirmed, additional bioinformatics analysis of ccRCC has not yet been performed. In this study, we first explored the mRNA expression and prognostic value (OS and PFS) of different cysteine cathepsins in ccRCC by using the GEPIA and Human Protein Atlas databases. The results showed that CTSF, CTSL and CTSO were associated with favorable prognoses in ccRCC patients. CTSK and CTSZ were associated with poor prognosis in ccRCC patients.

Interestingly, based on the above expression and prognostic results for cysteine cathepsins, we found that the mRNA and protein expression levels of CTSF were apparently downregulated in ccRCC tissues and closely related to the poor prognosis of patients with ccRCC. Moreover, our cell experiments proved that the overexpression of CTSF significantly suppressed the proliferation and migration of 786-O and 769-P cells. These findings suggested that CTSF might be a potential therapeutic target for the management of ccRCC proliferation and migration. Additionally, we used Gene Set Enrichment Analysis(GSEA) to elucidate the possible pathways influenced by CTSF, and found that CTSF co-expressed genes were associated with cell cycle, NOD-like receptor signaling pathway, p53 signaling pathway, NF-kappa B signaling pathway, etc. (Supplementary Fig. S4). Future experiments are needed to focus on elucidating the exact pathways by which CTSF affects proliferation and migration in ccRCC cells.

To gain more insight into the underlying molecular mechanisms CTSF in ccRCC, we further identified the co-expressed genes using GeneMANIA, and then performed GO and KEGG enrichment analysis used DAVID software on co-expressed genes. The results show that CTSF and its related proteins were involved mainly in the immune response, antigen processing and presentation, and the adaptive immune response. Previously, studies have revealed that CTSF is capable of processing the invariant chain that plays an important role in major histocompatibility complex (MHC) class II. The inhibition of cathepsin F activity could block MHC class II processing in macrophages^26,27^. Moreover, CTSF have been reported to associate with specific immune cells and immunomodulators. For example, CTSF expression was correlated with that of immune cell molecular markers and immunomodulators both in lung adenocarcinoma (LUAD) and lung squamous cell carcinoma (LUSC)^14^. However, the role of CTSF in the immune microenvironment in ccRCC has not been determined. In this study, we found for the first time that CTSF expression was significantly associated with tumor-infiltrating lymphocytes (TILs), immunoinhibitors, immunostimulators and MHC molecules in ccRCC. First, our study showed that the expression of CTSF was negatively correlated with the abundance of activated CD4 T cells (Act CD4), memory B cells (Mem B), immature B cells (Imm B), type 2 T helper cells (Th2), eosinophils and effector memory CD8 T cells (Tem CD8^+^ cells) in ccRCC. In addition, CTSF expression was negatively correlated with the expression of several immunoinhibitors (including CTLA4, TIGIT, CD96, LAG3, CD274, BTLA, PDCD1LG2 and PDCD1), immunostimulators (including ICOS, CD80, TNFRSF9, IL2RA, CD28, LTA, TNFSF13B, MICB, TNFSF4, TNFSF14, KLRK1 and CD86), and MHC molecules (including TAP1 and TAP2) in ccRCC. Published work have shown that high lymphocyte infiltration and immunosuppression characterized the immune microenvironment in ccRCC and contributed to cancer development^28,29^. Infiltration of CD8^+^ T cells within the tumor microenvironment (TME) may be associated with a poor prognostic marker and may predict the response to immune checkpoint blockade (ICB) in ccRCC^28^. Interestingly, our present study found that CTSF was downregulated in ccRCC tissues and negatively correlated with lymphocyte infiltration, which was similar with it. These findings suggested that CTSF potentially functions to regulate the tumor immune microenvironment in ccRCC and contribute to cancer development, but further studies are needed to explore the molecular mechanism involved in the regulation of the tumor microenvironment.

The present study improves our understanding of the relationship between Cathepsin F and clear cell renal cell carcinoma, but some limitations still exist. First, the sample sizes varied across different databases using in the present study, and were determined to be sufficient for detecting the reported differences for the standardized effect sizes at medium (d = 0.5) base on the G*power sample size software^30^. However, its not sufficiently powered to detect a small effect (d = 0.2), and a well-designed and large-scale clinical study would strengthen the reliability of the findings. Second, as the retrospective nature of the data analysis in the present study, further functional studies were needed to establish causality between CTSF expression and ccRCC progression. Third, despite the use of multiple bioinformatics databases in our study, independent validation of the CTSF expression levels and its impact on survival using additional patient cohorts, ideally from different populations and institutions, would greatly bolster the generalizability of the findings. Fourth, we observed that the overexpression of CTSF suppressed the ccRCC cell proliferation and migration by cell experiments. However, a deeper exploration of the underlying molecular mechanisms would strengthen the study, and future experiments could focus on elucidating the exact pathways by which CTSF affects proliferation and migration in ccRCC cells. Besides, we need further research to explore CTSF-targeted therapies and their potential synergism with existing treatments for ccRCC.

In conclusion, our study comprehensively analyzed the expression and prognostic value of different cysteine cathepsin family members in ccRCC and revealed that CTSF could be an ideal therapeutic target and a promising diagnostic marker in ccRCC. The expression level of CTSF was apparently downregulated in tumor tissues and closely related to the poor prognosis of ccRCC patients. Moreover, CTSF may contribute to the regulation of the tumor immune microenvironment in ccRCC. Our study could lead to the identification of new diagnostic and prognostic biomarkers and therapeutic targets for ccRCC.

## Materials and methods

### The University of ALabama at Birmingham CANcer data analysis (UALCAN)

UALCAN (http://ualcan.path.uab.edu/) is an interactive web resource for analyzing cancer OMICS data and includes the Cancer Genome Atlas (TCGA), MET500, Clinical Proteomic Tumor Analysis Consortium (CPTAC) and Children Brain Tumor Tissue Consortium (CBTTC) datasets^31^. In the present study, the mRNA and protein expression levels of different cysteine cathepsin family members in kidney renal clear cell carcinoma (KIRC) primary specimens were compared with those in normal controls. Z-values represent standard deviations from the median across samples for the given cancer type. Log2 Spectral count ratio values from CPTAC were first normalized within each sample profile, then normalized across samples.

### Tumor immune estimation resource (TIMER)

TIMER (https://cistrome.shinyapps.io/timer/) is an interactive online platform for the systematic analysis of immune infiltrates across diverse cancer types^32^. In this study, the expression of different cysteine cathepsin family members in KIRC primary specimens was compared with that in normal controls. Distributions of gene expression levels were transformed to log2 PTM values, with statistical significance of differential expression evaluated using the Wilcoxon test.

### Gene expression profiling interactive analysis (GEPIA)

The GEPIA database (http://gepia2021.cancer-pku.cn/) is an interactive online platform for analyzing gene expression in tumor and normal tissue samples from The Cancer Genome Atlas (TCGA) and Genotype-Tissue Expression (GTEx) databases^33^. In this study, we used GEPIA to generate survival curves for overall survival (OS) and disease-free survival (DFS) by classifying the KIRC patients into two groups by median expression (high vs. low expression) of different cysteine cathepsin family members. The *p* value was calculated using the logrank test and the cox proportional hazard ratio (HR) and the 95% confidence interval information were included in the survival plot.

### The human protein atlas

The Human Protein Atlas (https://www.proteinatlas.org/) is an interactive online platform that maps all human proteins in cells, tissues, and organs via the integration of various omics technologies^34^. In this study, we explored the prognostic value of different cysteine cathepsin protein-coding genes by classifying the KIRC patients into two groups by the best cut-off value (high vs. low expression). The best expression cut-off refers the gene expression value that yields maximal difference with regard to survival between the two groups at the lowest log-rank *P*-value.

### GeneMANIA

GeneMANIA (http://genemania.org) is an interactive online platform for generating hypotheses about gene function, analyzing gene lists and prioritizing genes for functional assays^35^. In this study, we constructed a gene‒gene interaction network for CTSF and the 20 genes related to the genes of interest to evaluate the potential functions of these genes. Categories are displayed up to a Q-value cutoff of 0.1.

### Database for annotation, visualization, and integrated discovery (DAVID)

DAVID (https://david.ncifcrf.gov/summary.jsp) is an online platform that provides a high-throughput and integrated data-mining environment for analyzing gene lists derived from high-throughput genomic experiments^36^. In this study, the functions of CTSF and the 20 most related genes were predicted via analysis of gene ontology (GO) and Kyoto Encyclopedia of Genes and Genomes (KEGG) data in DAVID against the background of *Homo sapiens*. All result of Chart Report has to pass the thresholds (Max.Prob. ≤ 0.1 and Min.Count ≥ 2) in Chart Option section to ensure only statistically significant ones displayed.

#### TISIDB

TISIDB (http://cis.hku.hk/TISIDB/) is a web portal for tumor and immune system interaction information that combines various heterogeneous data sources^37^. The database selected immune-related signatures of 28 immune cell types from Charoentong’s study. For each cancer type, gene set variation analysis (GSVA) package was used to identify genes related to abundance of tumor infiltrating lymphocytes (TILs) using gene expression profile of patients.

In this study, we explored the relationships between CTSF and TILs, immune inhibitors, immunostimulators and major histocompatibility complex (MHC) molecules in ccRCC.

### Cell culture and plasmid construction

786-O and 769-P cells were obtained from iCell Bioscience, Inc. Co., Ltd. (Shanghai, China). The cells were cultured in RPMI-1640 (Gibco) medium supplemented with 10% FBS. All cells were maintained in a humidified cell incubator with 5% CO_2_ at 37 °C. The method for plasmid construction and transfection was performed as previously described^38^. CTSF was cloned and inserted into the pCMV-HA vector and identified by sequencing.

### Cell proliferation assay

Cell proliferation was assessed as previously described^38^. In brief, 786-O and 769-P cells were seeded in a 6-well plate at 4 × 10^4^ cells/well. The cells in one of the 6 wells were digested with trypsin–EDTA (0.25%) at 37 °C the next day. The cell pellets were collected by centrifugation (1000 rpm for 5 min), washed with PBS, resuspended in PBS, and then counted under a microscope. Similarly, after three days, the cells were counted via a similar method.

### Migration assay

Cell proliferation was assessed as previously described^38^. In brief, 786-O and 769-P cells were seeded in 24-well cell culture inserts (BD, 353,097). After 48 h, the cells were fixed with 4% paraformaldehyde and stained with 0.5% crystal violet, after which the migrating cells were counted.

### Western blotting

Western blotting was performed as previously described^39^. The anti-Cathepsin F (AF2075-SP, dilution: 1:2000) antibody was purchased from R&D Systems. The anti–actin (A1978, dilution: 1:5000) antibody was purchased from Sigma‒Aldrich.

### Statistics

For the cell proliferation and migration experiments, all the data were analyzed with GraphPad Prism 9.3. Each experiment was independently performed three times, following the principle of repeatability. Differences between two groups of data were analyzed by Student’s *t* test. *P < 0.05; **P < 0.01. *P < 0.05 was considered to indicate statistical significance.

### Supplementary Information

Supplementary Information.

## Data Availability

The datasets analyzed in this study are available in the following repository: UALCAN (http://ualcan.path.uab.edu/), TIMER (https://cistrome.shinyapps.io/timer/), GEPIA (http://gepia2021.cancer-pku.cn/), the Human Protein Atlas (https://www.proteinatlas.org/), GeneMANIA (http://genemania.org), DAVID (https://david.ncifcrf.gov/summary.jsp), TISIDB (http://cis.hku.hk/TISIDB/). Other data used to support the findings of this study are available from the corresponding author upon request.
