# Supplementary material for: Reduced expression of cathepsin F predicts poor prognosis in patients with clear cell renal cell carcinoma
Source: Sci Rep. 2024 Jun 12;14:13556. doi: 10.1038/s41598-024-64542-2 (PMC11169360; doi:10.1038/s41598-024-64542-2)
Supplement: Supplementary file 1 — Supplementary Information. [file 41598_2024_64542_MOESM1_ESM.pdf]

Reduced Expression of Cathepsin F Predicts Poor Prognosis in Patients with Clear Cell Renal Cell Carcinoma

Xin Zhou,[1] Huayan Chen,[2] Dong Huang[2], Guixian Guan,[3] Xiaoli Ma,[2] Weiming Cai,[2]✉ Jing Liao,[4]✉ and Tangming Guan[2]✉

Supplemental Material.

Supplementary Figure 1.

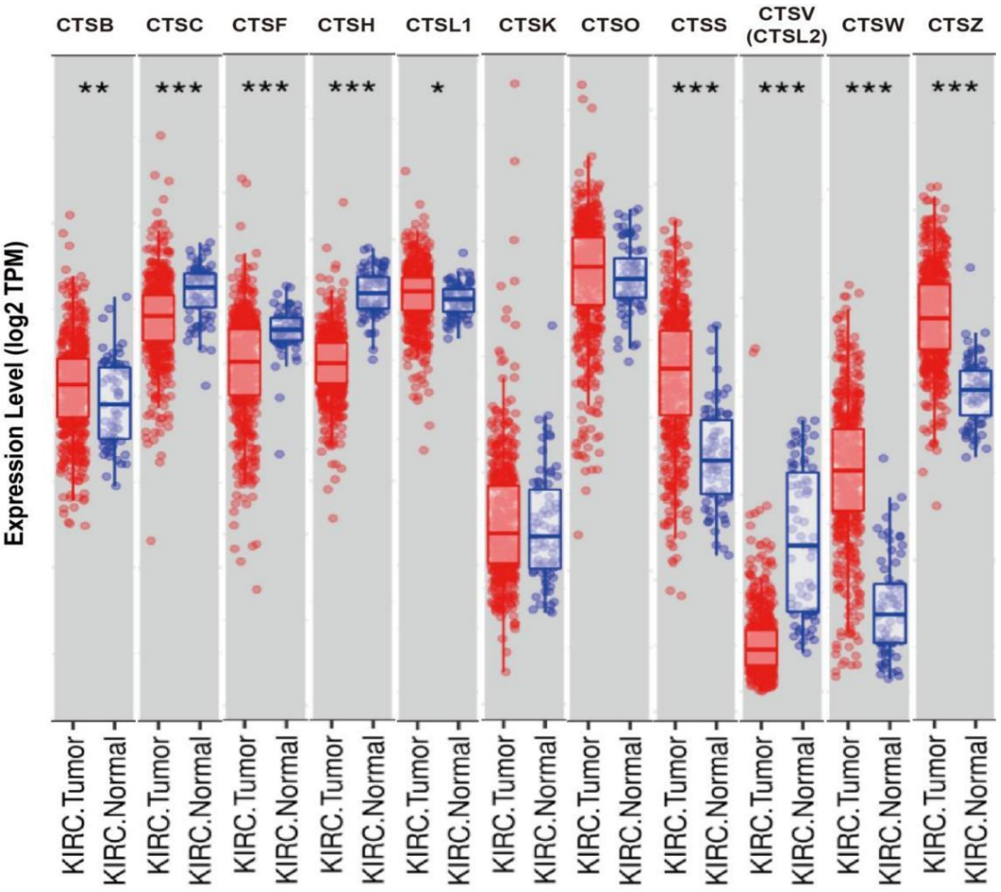

Supplementary Figure 1. mRNA expression levels of the cysteine cathepsin family members in KIRC and normal tissues according to the TIMER database. Red, primary tissues of KIRC. Blue, normal tissues. * $p < 0.05$, ** $p < 0.01$, *** $p < 0.001$.

Supplementary Figure 2.

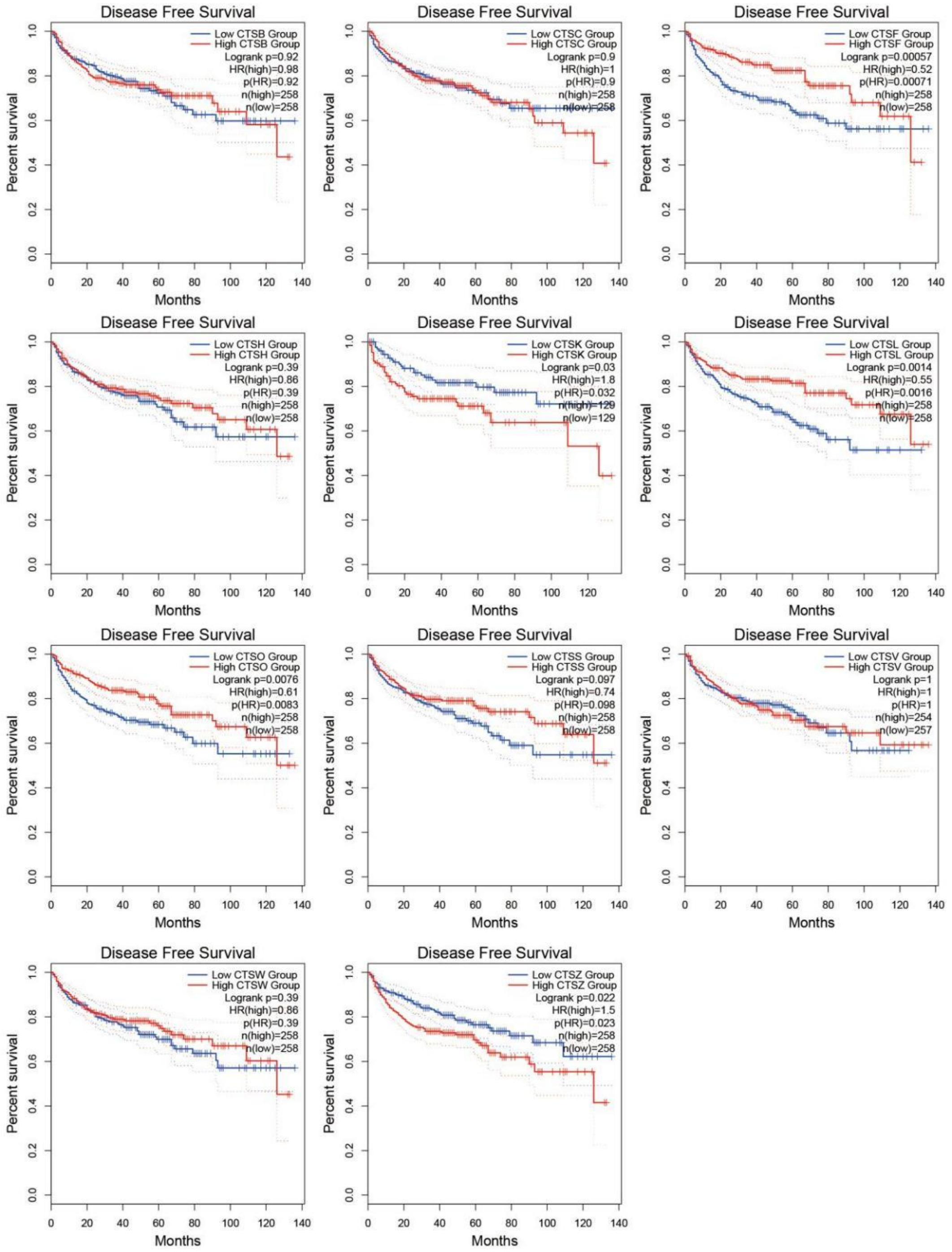

Supplementary Figure 2. The disease free survival (DFS) of KIRC patients based on the cysteine cathepsin family genes mRNA expression according to the GEPIA database. HR refers to hazard ratio. Data were analyzed using the Kaplan-Meier Plotter.

Supplementary Figure 3.

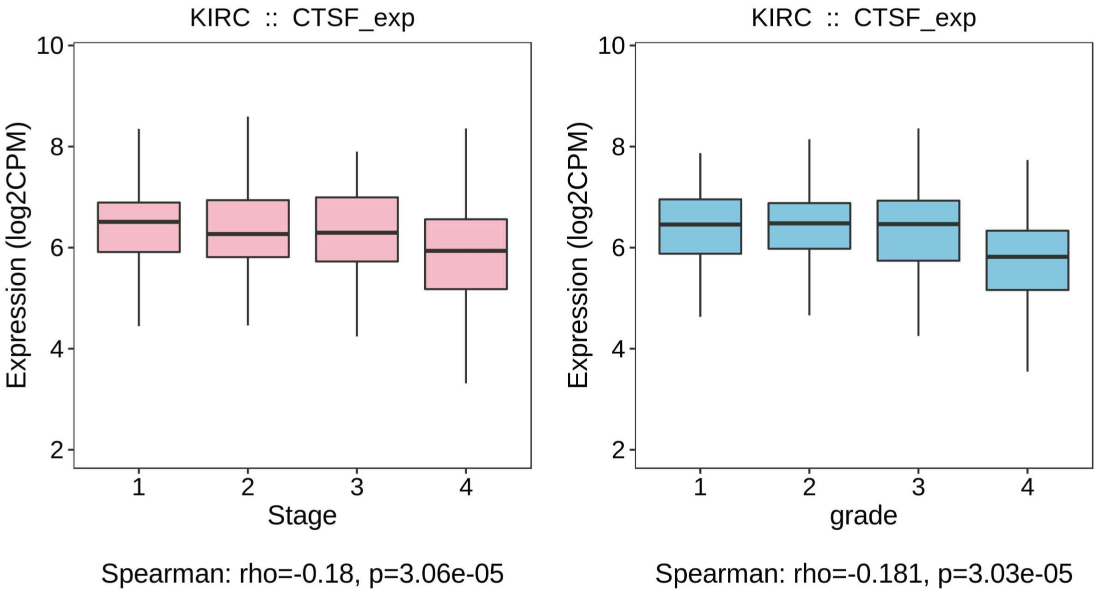

Supplementary Figure 3. The relationship between the mRNA expression levels of different CTSF and clinical cancer stages and grades in patients with KIRC according to the TISIDB databases.

Supplementary Figure 4.

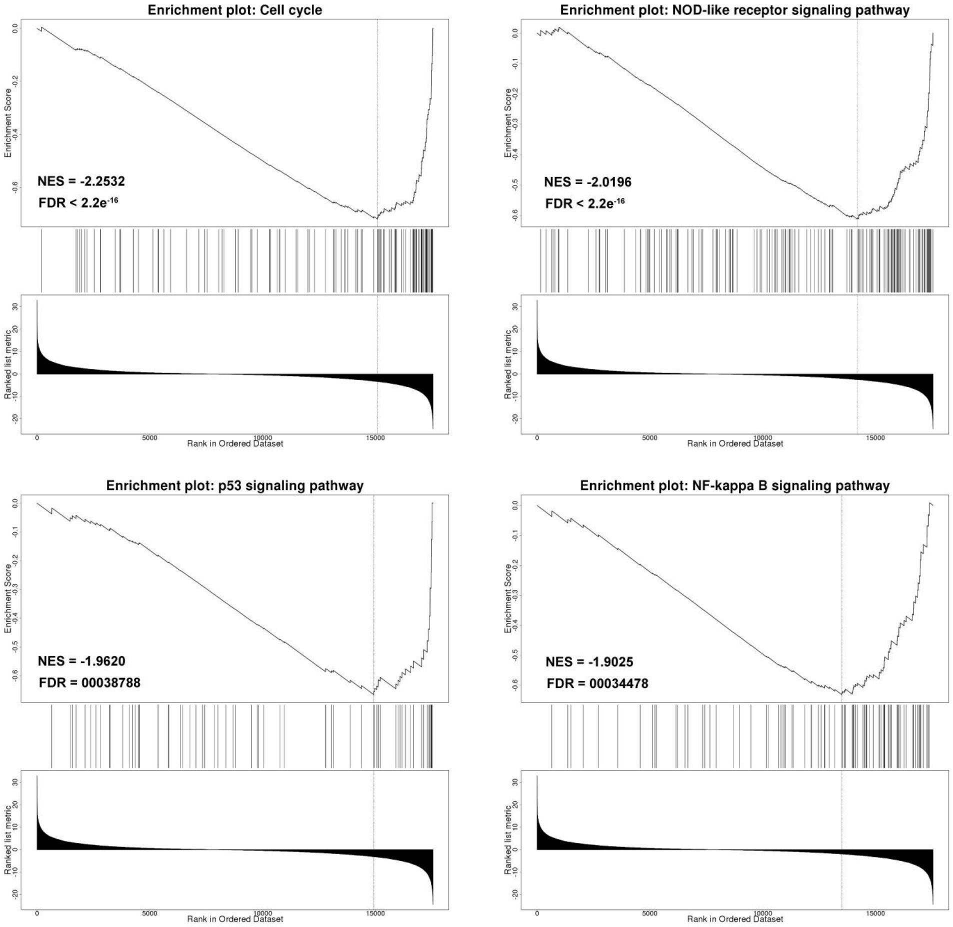

Supplementary Figure 4. The main enriched KEGG pathways of CTSA according to the LinkedOmic databases. CTSF co-expressed genes were associated with Cell cycle, NOD-like receptor signaling pathway, p53 signaling pathway and NF-kappa B signaling pathway.

Supplementary Figure 5.

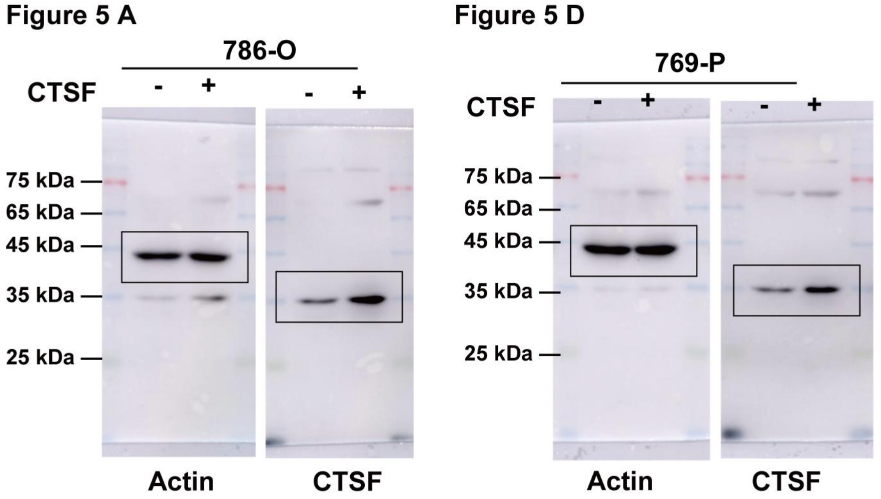

Supplementary Figure 5. Original scan of the blots presented in the main text.

Related to Figure 5.
